# COVID-19, mobility restriction, and sexual behavior among a cohort of people of reproductive age: Nigeria

**DOI:** 10.3389/fpubh.2022.935625

**Published:** 2023-01-09

**Authors:** Paul O. Adekola, Favour C. Ugwu, Emmanuel O. Amoo, Adebanke Olawole-Isaac, Giuseppe T. Cirella

**Affiliations:** ^1^Covenant University, Ota, Nigeria; ^2^University of Gdansk, Sopot, Poland

**Keywords:** lockdown, sexual well-being, social development, socio-demographics, COVID era, Owerri, South-East

## Abstract

**Background:**

One of the non-pharmaceutical strategies adopted by various governments to control the spread of COVID-19 is mobility restriction (MR), popularly known as a lockdown. Evidence shows that MR has some unintended consequences, such as increased cases of domestic violence, rape, pornography, sex chats, incest, and other unhealthy sexual behaviors (SBs).

**Methods:**

The study examined the influence of MR on SB in Owerri *via* a cross-sectional quantitative approach. A total of 425 interviewees were randomly chosen among people of a reproductive age. Data were analyzed using univariate, bivariate, and multivariate levels of analysis.

**Results:**

A significant relationship exists between selected socio-demographic characteristics, such as age and marital status, and the ability of people to cope with sexual abstinence. Results from the logistic regression analysis further illustrated this observation as during MR people were twice as likely to engage in prolific sex chats that could spur other harmful SBs.

**Conclusion:**

It is recommended that people should be allowed to determine whether they would like to stay with their partners in subsequent lockdowns, or otherwise, to prevent some of the unpleasant SBs recorded.

## 1. Introduction

In December 2019, a novel coronavirus, later reported as COVID-19, emanated from Wuhan, from where it spread to other regions of China and the world (1–3). The COVID era has wreaked serious havoc on business and industry across most nations (4–6). The goal of governments and public health practitioners has been to protect people from contracting COVID-19 and control its spread. One of the non-pharmaceutical strategies adopted is mobility restriction (MR), popularly referred to as a lockdown (7–12). While the strategy yielded appreciable results in controlling the spread of COVID-19 in some places (9, 13–17), studies are emerging about the unintended damaging health consequences. There are reports about cases of intimate partner violence (IPV), domestic violence, rape, incest, pornography, and other unhealthy sexual behaviors (SBs) in many societies where lockdown was judged to be effective (18–21). It is an old yet still relevant fact that diseases mostly spread through migration, as evidenced in past pandemic events, even before this century (22). For instance, studies show that the Black Death most likely originated in Central Asia, from where it traveled along the Silk Road, reaching Crimea by 1,347 through trading routes (23, 24). From there, it was most likely carried by fleas living on the black rats that traveled on Genoese merchant ships, spreading throughout the Mediterranean and reaching Africa and the rest of Europe *via* Constantinople, Sicily, and the Italian Peninsula (23–25). Hence, economic activities between countries, subregions, regions, and even continents in the name of progress and globalization can aid the global spread of infectious diseases (22, 26). COVID-19 is not an exception. The virus was brought to Nigeria from Italy in February 2020 and quickly spread to other states before the federal government announced a total nationwide lockdown on 20 March 2020 to curtail its spread (27). Although each region in the country had control over when to relapse lockdown rules, the lockdown generally lasted for more than 6 months across the country.

However, as good as the intention of MR was, studies have reported that it has the potential to induce rape, domestic violence, unintended pregnancy, abortion, multiple sexual partners, and other unhealthy SBs, not only in Nigeria but all around the world (7, 28–34). SBs have been described as various conducts and activities that are intended to arouse the sexual interest of oneself or another (35). Studies have confirmed that there are certain healthy SBs while a few reports have also condemned certain SBs, such as some of those described as inimical to healthy living (35, 36). However, some unhealthy SBs, which were commonly reported during the peak of Nigeria's MR period, included IPV, pornography, masturbation, sex chats, rape, and incest (20, 21). While it is expected that these unhealthy behaviors could be rampant in regions or states where there is already a high prevalence of rape, unintended pregnancy, IPV, and so on, there is a great deal of worry about the silence of SB in states seemingly unknown for these negativities. Although there is a low prevalence of risky SBs in certain states, a key inquiry intends to find out what happened during the COVID-19 lockdown? To answer this, the emerging nexuses between MR and risky SBs must be investigated. As a result, the major aim of this work is to examine the effects of MR on SBs among the population of reproductive age who were locked down in Owerri, Imo State, during the peak of COVID-19 in the country. Findings from this study could provide insight into the sources of unintended pregnancy and sexual violence, and spur adequate interventions from government, public health practitioners, social workers, and other stakeholders in sexual well-being. The results and viewpoint of the study should aid regional and national representatives in better exploring the issue and further promote debate and in-depth analyses in terms of public health. Only limited studies of this kind have been published in Africa, making this study an important step that complements the nexus between mobility and sexuality.

## 2. Methods

### 2.1. Research design and sample size

**Figure 1:**
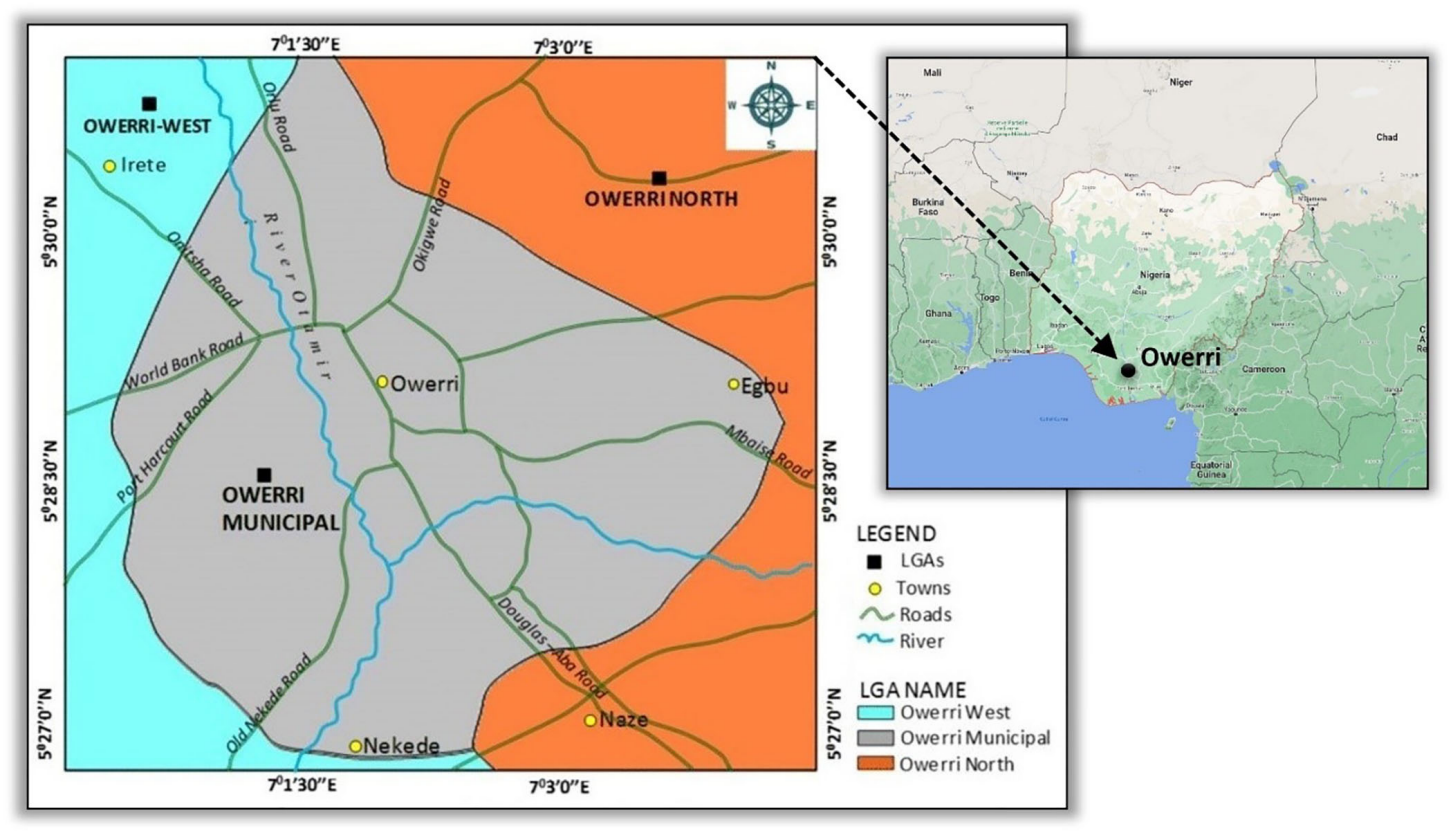
Location of the study area, adapted from Google Maps 2022.

This study adopts a cross-sectional quantitative research design using a questionnaire as its primary method of data collection (see Supplementary material 1 for a sample of the questionnaire used). The participants included male and female cohorts of reproductive age (i.e., 15–44 years old) from the city of Owerri in South-East, Nigeria. The questionnaire was administered during the period of the COVID-19 lockdown in Nigeria. Participants of the study did not necessarily have to be permanent residents of the city, only that they were “locked down” in it during the fieldwork. The reproductive age cohort was purposively chosen due to their high probability of being sexually active or in a romantic relationship. Hence, this research is concerned about understanding how residents of Owerri in their reproductive years reacted sexually to MR and whether this was the first time they experienced MR, as well-determining whether their partner was away from the vicinity during lockdown and, if so, what action was taken. The decision to survey Owerri was based on the personal experience of the second author who was stranded in the city during the lockdown and was able to conduct the questionnaire in real time. The research took place during a 7-month lockdown from March 2021 to September 2021. Owerri is the capital city of Imo State, and one of the most densely populated cities in South-East, Nigeria. Owerri is inhabited by the Ibo people, the majority of whom are Christians and speak Igbo as a major language. Imo State is the 14th most populous state in Nigeria and has the lowest adolescent pregnancy rate among the 36 states of the country (37, 38). The state had an adolescent fertility ratio of 14 per 1,000 live births in 2015 (37, 39) and this has remained consistently low. Owerri is officially made up of three local government areas: Owerri Municipal, Owerri North, and Owerri West. For this study, only Owerri Municipal, the main township, was considered (Figure 1). In 2006, the population was 127,213 (61,682 males and 65,531 females), according to the national population and housing census (39). This is the last known population size of Owerri Municipal. For this study, a projected population for 2020 was calculated. Noting the rapid population growth rate in sub-Saharan Africa and Nigeria, i.e., an annual growth rate of 2.5% (40, 41), the 2020 projection yielded a population of 707,900 for the five main local government areas of the state using Equation 1.


(1) $$\begin{array}{l} P_{t}=\;{P_{0}\left(1=r\right)}^{n}, \end{array}$$

where *P*_*t*_ is the projected population, *P*_0_ is the base population, *r* is the growth rate, and *n* is the number of years between the two periods (i.e., 14 = 2020-2006).

Based on this projection, the population of Owerri Municipal in 2020 is therefore approximately 306,922. Applying “the rule of 70” (i.e., 70 divided by the annual growth rate), Owerri Municipal would double its present population in the next 28 years (i.e., in 2048). With the target population calculated, Phrasisombath's (42) sample size formula for a known population was adopted using Equation 2.


(2) $$\begin{array}{l} n\;=\;\frac{NZ^{2}{\alpha /}_{2}\;P\left(1-P\right)}{e^{2\;}\;\left(N-1\right)\;+\;NZ^{2}{\alpha /}_{2}\;\left(1-P\right)\;}, \end{array}$$

where *n* is the sample size, *N* is the target population, *P* is the estimated proportion of the population that represents the characteristics, *Z*^2^α/2 is the level of statistical significance according to a standard normal distribution, and *e* is the tolerated margin error.

Since the target population is known, as it is in this case, the level of confidence is 95%, which conventionally equates to *Z* = 1.96. In terms of the level of precision, *e* is considered 0.01, which should produce a high level of precision and small error of estimation. Using this method, the generated initial sample size was 384. However, we allowed for an additional 10% of tolerance due to a suggested high non-response rate and adjusted the sample size to 422.

Owerri Municipal is divided into five administrative divisions. A random proportional sampling method was used to select the sample size from each as they differ in population size (Table 1). Each respondent was given the option to remain anonymous and their responses were treated with strict confidentiality. The respondents were also given information about the intent and context of the study. They were also kept informed about the process and participation procedure. The questionnaire focused on piecing together information on the effects of MR on the SBs of people of reproductive age in Owerri. The questionnaire was subdivided into two sections: section A asked for socio-demographic information and section B probed on the effects of MR on SB. Section B focused on common SBs during the COVID-19 lockdown, as described in the SB-oriented literature (43–48).

**Table 1:** Determination of sample size.

**Administrative divisions**	**2006 population**	**2020 population (projected)**	**Proportion of sample size (%)**	**No. of distributed questionnaires**
Umuororonjo	70,000	100,000	14	59
Amawom	150,000	197,900	27	114
Umuonyeche	101,000	160,000	23	97
Umuodu	80,000	110,000	16	68
Umuoyema	100,000	140,000	20	84
Total	501,000	707,900	100	422

### 2.2. Psychometric analysis: Reliability and validity

Two specific tests were used to make up the psychometric analysis, namely, reliability and validity. Reliability answers the question on whether the instrument can yield consistent information if it is applied to the target audience on repeated occasions (49). This is conducted using Cronbach's alpha coefficient and is one of the most common methods of checking internal consistency for reliability. The test was carried out using the software package SPSS version 23 with data obtained from the completed questionnaires. The normal range for Cronbach's alpha coefficient for reliability is between 0 and 1 (49). The reliability of the data was accepted as the return value equated to 0.84.

Validity is important because the research instrument is defined as the degree to which the items in an instrument reflect the content of the study, as stated by Tavakol and Dennick (50). For the validity test segment, only face validity was used. For this test, additional experts (i.e., specialists) in quantitative survey appraisal assessed the instrumentation as suitable, concluding that the analysis, if conducted in its entirety, has the potential to measure what it desires. The appraisal was logically linked to the research objective and based on the respondents' feedback before a decision was made.

### 2.3. Data analysis

The study utilized three levels of data analysis: univariate, bivariate, and multivariate statistical techniques. Univariate statistical tools were used to present the respondents' background characteristics using percentage distribution and tables. The bivariate analysis examined the relationship between selected socio-demographic characteristics and sexual abstinence during the COVID-19 lockdown in the absence of a spouse. For this part of the study, the basic statistical tool used was the chi-squared test. The multivariate analysis examined the influence of MR on SB. The dependent variable was measured as a binary code (i.e., no = 0 and yes = 1), making the response variable amendable to binary logistic regression. Binary logistic regression was used to test the odds of unhealthy SBs being influenced by MR. The ability to move about equated to either “no” or “yes”. Binary logistic regression is used to help predict the likelihood of a person engaging in unhealthy sexual gratification because of the inability to move about (probably) to places where these can be granted. A response of “yes” was set as the reference category (RC) from where other responses were referenced. Conversely, the independent variables are SBs, which were defined as behavior and activity that are intended to arouse the sexual interest of oneself or another (35). Different dimensions of SBs have been reported throughout the COVID era in many countries throughout the world. However, eight prominent SBs from the literature formulated the variables of the study and stood out as especially concerning, including pornography, sending nude pictures, rape, masturbation, watching romantic movies, sex chats, and incest. The analysis adopted a 0.05 level of significance, which gave a 95% confident interval on the assurance of accepting or rejecting the null hypothesis. All recovered data from the field were coded and analyzed using SPSS version 23. The general model for the binary logistic regression utilized the calculation for the likelihood of being able to move about during the lockdown (i.e., by checking for the implicit function of the hypothesis), the specific variables presented in the study, and the explicit function of the binary logistic regression formulation against the independent variable (see Supplementary material 2 for a detailed breakdown of the formulation used for the data analysis).

## 3. Results

### 3.1. Socio-demographic characteristics

A total of 98.8% of the interviews were successful, which equaled an attrition rate of 1.2%. This was a result of the lockdown measures, which meant virtually everyone was available. Table 2 shows the distribution of the respondents in terms of socio-demographic characteristics. Relatively, half of the respondents (i.e., 54.4%) reside in the location of the study while others were made up of visitors or migrants from other states. In terms of state of origin, other key states that stood out were Enugu State (i.e., 8.2%), Anambra State (i.e., 10.6%), Ebonyi State (5.2%), and Abia State (10.1%). As the study took place in South-East, we were also interested in those who participated in the study but were not originally from this part of the country; they represented 11.5%. This implies these participants were from either the western or northern states, or foreigners. The results in terms of ethnicity show that the majority (85.5%) are Igbo. Yoruba ethnic stock represented 7.5% of the respondents while the Hausas made up 4.9%. Again, the others, which accounted for 2.1%, were probably foreigners. The univariate result also shows that most of the respondents (80.7%) had tertiary education, which corresponds with Owerri's status as a multiple university city.

**Table 2:** Distribution of respondents by socio-demographic characteristics.

**Socio-demographic characteristics**	**Frequency**	**Percentage**
	**(*N* = 425)**	
**State of origin**		
Imo	231	54.4
Enugu	35	8.2
Anambra	45	10.6
Ebonyi	22	5.2
Abia	43	10.1
Others	49	11.5
**Ethnicity**		
Igbo	363	85.5
Hausa	21	4.9
Yoruba	32	7.5
Others	9	2.1
**Level of education**		
No formal education	21	4.9
Primary education	8	1.9
Secondary education	53	12.5
Tertiary education	343	80.7
**Age group**		
15–24	211	49.6
25–34	175	41.2
35–44	39	9.2
Total	425	100.0
**Gender**		
Female	241	56.7
Male	184	43.3
Total	425	100.0
**Marital status**		
Single	314	73.9
Married	100	23.5
Divorce	4	0.9
Widowed	1	0.2
Never married	6	1.4
**Number of months lockdown lasted**		
1–3 months	19	4.5
4–6 months	210	49.4
7–9 months	164	38.6
10 months and above	32	7.5

Authors' own computation from the field survey, 2021.

Approximately 5% of the participants had no education at all, while 12.5% and 1.9% had secondary and primary education, respectively. The age distribution shows that most of the respondents belonged to the age cohorts of 15–24 years old (49.6%) and 25–34 years old (41.2%), while 9.2% of them belong in the 35–44-years-old cohort. Results from the gender distribution showed more female participants (56.7%) than male participants (43.3%). Moreover, most of the respondents were single (73.9%) as per marital status, while 23.3% were married. Other marital categories include those who were divorced (0.9%), widowed (0.2%), and those who were never married but have partners with whom they cohabitate (1.4%). The last but one of the most important results in the univariate analysis was to ask participants how long they felt the MR lasted in Owerri. Those who believed the MR lasted between 1–3 months were 4.5%, while 49.4% of them said that the MR lasted from 4–6 months. On the longer side, approximately 39% of them said it lasted for 7–9 months and, surprisingly, 7.5% of them went on to claim that they had been in lockdown for more than 10 months, which was false as the government mandate was only 7 months.

### 3.2. Bivariate analysis interrelating selected background characteristics and sexual abstinence

The bivariate analysis was conducted using cross tabulation and the chi-squared test to examine the relationships between selected socio-demographic characteristics and the ability to endure sexual abstinence during the COVID-19 lockdown. The results showed that there were significant relationships between some selected socio-demographic variables and the ability to endure sexual abstinence (Table 3). Four socio-demographic variables, i.e., age, religion, gender, and marital status, were cross-tabulated against sexual abstinence during the lockdown. The age and religion affiliations were significantly related to sexual abstinence during COVID-19 lockdown (i.e., chi-square = 18.997; *p* < 0.05). Approximately 42% of the respondents aged 15–24 years old abstained from sexual activities during the lockdown, while 58.3% did not. A similar result was obtained for those aged 25–34 years, as 46.9% of them were able to endure sexual abstinence while 53.1% could not. Only in the older age group (i.e., 35–44 years old) were there more adults who claimed they were able to hold up sexually during MR (66.7%) than those who claimed they could not (33.3%). These results are very telling as they imply some range of probability of sexual violence and unhealthy SBs relative to age. This is stated only in terms of the probability and will be further explored in the multivariate analysis.

**Table 3:** Percentage distribution of selected background characteristics and ability for sexual abstinence during the COVID-19 lockdown.

**Selected variables**	**Sexual abstinence during lockdown**		**Chi-squared**	***p*-value**

	**No**	**Yes**		
**Age group**				
15–24	123 (58.3%)	88 (41.7%)		
25–34	93 (53.1%)	82 (46.9%)		
35–44	13 (33.3%)	26 (66.7%)	8.318	0.010^*^
**Religion affiliation**				
Christianity	213 (53.7%)	184 (46.3%)		
Islam	11 (4.8%)	12 (6.1%)		
Traditional	5 (2.2%)	—	4.627	0.038^*^
**Gender**				
Female	138 (46.9%)	103 (53.1%)		
Male	91 (38.6%)	93 (61.4%)	2.558	0.067
**Marital status**				
Single	175 (44.7%)	139 (44.3%)		
Married	47 (47.0%)	53 (53.0%)		
Divorced	3 (75.0%)	1 (25.0%)		
Never married	3 (50.0%)	3 (50.0%)	3.494	0.359

Authors' own computation from the field survey, 2021; ^*^p < 0.05; chi-squared = 18.9974.

It is also important to stress that the results from marital status show that more single respondents (55.7%) claimed they were not able to maintain sexual abstinence during COVID-19 MR than their counterparts who were (44.3%). It is also interesting to note that the majority of divorcees (75.0%) said they could not maintain sexual abstinence during COVID-19 lockdown while those who were never married but cohabiting shared the statistics of sexual abstinence equally (50% / 50%). However, more of the respondents who were married (53.0%) and were living with their spouses said they were able to maintain sexual abstinence during MR than those who could not (43.0%). It is also interesting that more Christians (53.7%) were not able to maintain sexual abstinence during MR than Muslims (4.8%), although the majority of the respondents were Christians, which may have skewed the result. Surprisingly, more males (61.4%) were able to maintain sexual abstinence during COVID-19 MR than females (i.e., 53.1%). Next, the bivariate results were utilized and cross-referenced to formulate the multivariate analysis and show potential correlations with problematic and unhealthy SBs.

### 3.3. Probability of unhealthy sexual behavior

The multivariate analysis illustrated the responsiveness of identified SB indicators to MR. This evaluation was based on the probability of associating unhealthy SBs instigated by MR (i.e., correlated with the time period the city was in lockdown). The selected SB indicators were regressed against MR (Table 4); they included pornography, exchange of nude pictures, sex chats, watching erotic movies, masturbation, rape, incest, and self sex. Although our correlation result shows a weak positive association between MR and SB (*R* = 0.141), our regression result shows a significant influence of MR on SB (i.e., *p* < 0.01). Results from the logistic regression further show that the people who did not enjoy mobility during the COVID-19 lockdown were approximately 2 times more likely to engage in prolific sex chats, which involves having erotic discussions with another partner, including sending explicit pictures to connect with the sexual emotions of the other person, mostly a boyfriend or girlfriend compared with their counterparts who were able to move about. Further results also indicate that those who were not able to move about during the lockdown were 1.3 times, 1.01 times, and 1.03 times more likely to watch erotic movies, engage in masturbation, and engage in rape, respectively, compared with their counterparts who were able to move about one way or another. Moreover, respondents who were not able to move about during the COVID-19 lockdown were 1.1 times more likely to engage in watching pornography than those who enjoyed mobility. Compared with the bivariate results, this further reiterates the fact that there are serious implications of MR as people experiencing these SBs are likely to do anything to satisfy themselves sexually.

**Table 4:** Logistics regression estimating the odds of unhealthy SBs instigated by MR.

**SB variable due to MR**	**Odds ratio (Exp. β)**	**Str. Error**	***p*-value**
**Pornography**			
Yes	RC	—	—
No	1.078	0.220	0.733
**Nude pictures**			
Yes	RC	—	—
No	0.820	0.218	0.363
**Sex chats**			
Yes	RC	—	—
No	1.834	0.285	0.033^**^
**Erotic movies**			
Yes	RC	—	—
No	1.321	0.321	0.386
**Masturbation**			
Yes	RC	—	—
No	1.012	0.292	0.967
**Rape**			
Yes	RC	—	—
No	1.029	0.221	0.898
**Incest**			
Yes	RC	—	—
No	0.846	0.225	0.456
**Self sex**			
Yes	RC	—	—
No	0.755	0.222	0.205
Log likelihood	544.333		
R-squared	0.023		
LR Chi-squared	9.912 (df = 11)		
Prob > Chi-squared	0.000		

Authors' own computation from the field survey, 2021; ^**^p < 0.05.

**Figure 2:**
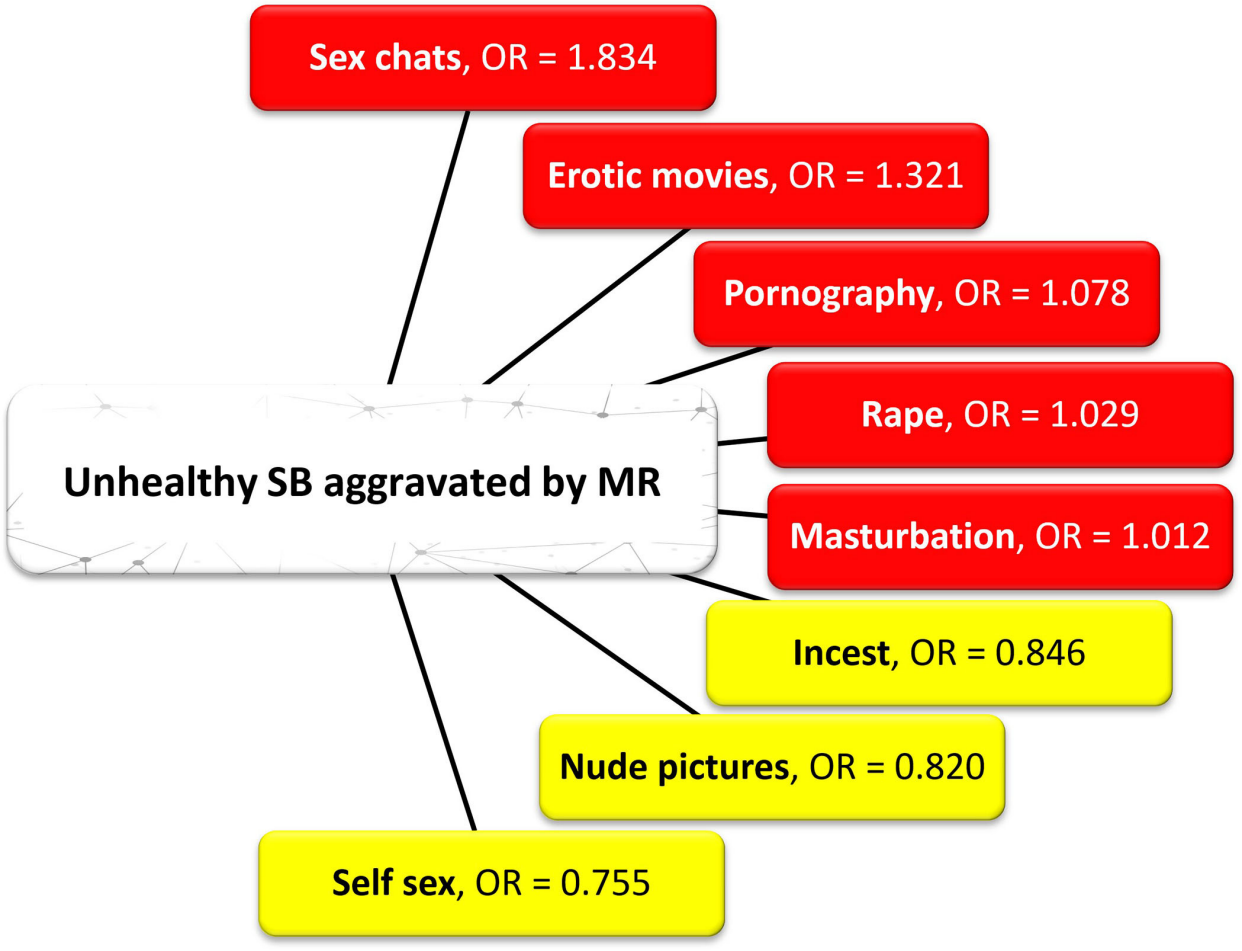
Unhealthy SB aggravated by the COVID-19 lockdown. According to the odds ratio (OR), the red-colored variables have a higher likelihood of occurrence vs. the yellow-colored variables which have a lower likelihood of occurrence.

A summary of the adjusted model shows that the dependent variable (i.e., MR) influenced SB to a tune of 2.3% in Owerri. This means that there was a shift of 2.3% in people unhealthily engaging in SB as they were not able to move about to get sexual satisfaction like they used to pre-lockdown. The result in the model shows a log likelihood ratio = 544.333, *R*^2^ = 2.3%, log chi-squared = 9.912 on 11 degrees of freedom, and *p* < 0.001. Therefore, it can be deduced that MR had a significant effect on unhealthy SBs in Owerri. The overall percentage from the regression analysis indicates a 63.5% level of accuracy in the outcome. The model summary shows a 2.3% and 3.2% level of variation in the predicted variable as explained by the independent variables given by the “Cox and Snell R squared” (i.e., 0.023) and “Nagelkerke R squared” (i.e., 0.032). This provides confidence that the model is relevant in demonstrating the effect of MR on SB. Overall, it can be stated that the statistical findings support the exercise of the model and its variable predictability. Figure 2 illustrates the unhealthy SBs that are worsened by MR, i.e., the red-colored variables indicate a higher likelihood of occurrence as interpreted by the odds ratio and the model, while those in yellow indicate a lower likelihood of occurrence.

## 4. Discussion

This study examined the exposure of a population of reproductive age to unhealthy SB during the COVID-19 lockdown in a Nigerian state that has been popularly censored or decorated as the least affected by unintended pregnancy and has low adolescent pregnancy rates (37). While shops, schools, markets, and other public places were closed and appropriate measures to prevent further escalation of the pandemic were put in place, the home fronts were seemingly let loose (i.e., unguarded), with high vulnerability of the population of reproductive age to rape, pornography, IPV, and other unhealthy SB. One of the highlights of the study indicated that approximately half of the population could not practice abstinence during the lockdown despite the non-availability (i.e., absence) of their spouse—although <25% of the studied population were married. This finding is a crucial signal to the degree of multiple sexual partnerships in the location and the enormity of the eventual consequences, such as an increase in the rate of sexually transmitted infections and the possibility of unintended pregnancies. Specifically, the study found that 58% of respondents aged 15–24 years could not practice sexual abstinence during lockdown. Additionally, more than half of the next higher-aged population (i.e., 25–34 years old, 53.1%) could not refrain from sexual activities during the period examined. These statistics indicate that the majority of the youth (i.e., 15–34) could not practice abstinence during the COVID-19 lockdown and may perpetually continue to act this way even without a lockdown. It is not impossible to insinuate that when the opportunity for consensual sex is limited or not available, rape or other sexual violence could be inevitable.

Increased cases of intimate partner violence, rape, partner beating, children and minor molestation, and other unhealthy SBs were reported in Botswana, Nigeria, Ethiopia, Italy, the United States, Australia, and others during the COVID-19 MR (18–20, 32–34). Tade (20), for instance, found that MR enhanced the tactics of sexual predators to sharpen their strategies and attack their victims in their neighborhoods—leading to increased rape cases and sexual victimization in Nigeria. Sex chats, watching erotic movies, masturbation, rape, and pornography are very dangerous, terrifying, and graphic SBs that, if practiced, can be enhanced during lockdown, especially with young girls (20). This may account for the five cases of gruesome rape described by Tade (20) in Nigeria, where girls in different locations throughout the country were involved, two of which, unfortunately, lost their lives in the process. There is a likelihood that some of the young men found to perpetrate these acts have been practicing some of the unhealthy SBs described in this study. Rape does not just occur without thinking about it and mapping out some strategy. Also, thinking about rape does not just occur without an individual who is practicing, in one way or another, other unhealthy SBs (51–54). Moreover, the study also found that people who did not enjoy mobility during the lockdown watched pornography. Literature has confirmed that most people who practice unhealthy SBs could be addicted to anything that satisfies their pleasure, including rape or other sexual violence (55, 56). However, while sexual violence has been erroneously regarded as a male affair, i.e., where males are seen as the main perpetrators (27), our study found that more women (46.9%) could not abstain from sex than men (i.e., 38.6%) during lockdown. However, the categories varied, with 75% being divorced women, and 50% of this total were cohabiting (see Supplementary material 3 for additional data on SB).

Besides MR, which may make the lodging of such occurrences difficult, the shame and intimidation from older women who rape young boys may contribute to low reporting. This is also supported by a study in Ghana that attributed a lack of social support and stigma as core reasons for the low reporting of IPV in that country (57). It is also worth noting that although IPV and unhealthy SBs may have been caused by MR, they have, however, been worsened by other associated stressors, such as loss of employment during the strict lockdowns, a greater burden of taking care of one's family, and the financial demands and stress this carries. This is especially dominant in the United States and Australia, where financial stress aggravated unhealthy SBs, such as IPV (32). As such, the process of implementing MR needs to be better understood. Governments should be aware of ways of catering for the sexual health and rights of those who will be locked down if any future occurrence demands MR. This will help curtail unintended consequences, such as increased cases of domestic violence, rape, incest, pornography, sex chats, and other unhealthy SBs, as found in this study. If this is not done, it might have an adverse implication on the fulfillment of Agenda 2,063, a 7-point agenda geared toward realizing a Pan-African vision of an integrated, prosperous, and peaceful Africa, driven by its own citizens and representing a dynamic force in the global arena (58). Not paying attention to peoples' sexual and reproductive rights and health may have a direct or indirect impact on the Agenda's Aspirations 3–6, which include an Africa of good governance, democracy, respect for human rights, justice and the rule of law, a peaceful and secure Africa, an Africa with a strong cultural identity, common heritage, values and ethics, and an Africa where development is people-driven, unleashing the potential of its women and youth (58). Moreover, we are in danger of not getting anywhere close to achieving the United Nations Sustainable Development Goals, specifically SDG 3, which is in place to ensure healthy lives and promote well-being for all ages (59, 60)—something that is not possible if cases of rape and other sexual violence continue in subsequent lockdowns.

It is also thought-provoking to note that at the peak of the COVID-19 pandemic, MR had far-reaching implications on behavior, lifestyle, and familial relationships *via* multiple announcements of variants, transmission information, and vaccination programs. There have been serious behavioral and lifestyle changes since it was announced that human-to-human transmission was possible and initial reports of animal-to-human transmission from the Wuhan seafood market may have played a part as the pandemic's epicenter (61–63). Direct human-to-human transmission from inhalation of droplets by way of coughing, sneezing, and saliva of infected persons was proclaimed as the manner in which COVID-19 spread (63, 64). This information partly explains the significant changes in hospital practice and behavior—especially for people seeking dental and optical care during the first 2 years of the pandemic. For example, personnel involved in dental care have been advised to be extra careful in the process of discharging their duties by wearing nose masks and hand gloves, while patients have also been compulsorily made to wear a nose mask. Similar social behaviors have been exposed to people with specialist contact lenses (CLs), although no scientific evidence shows any relationship between wearing or not wearing CLs and protection against COVID-19. Nonetheless, to reduce physical contact when visiting a health facility, people using CLs are advised to consider thorough daily cleaning of their lenses to avoid exposure of their CLs to water and using daily reusable lenses (65–67). On the family front, pregnant women have been considered to be at risk and have been advised to shun and avoid crowded locations or social gatherings, thereby effecting maternal and neonatal morbidity rates during the peak of the pandemic (64). Moreover, habits such as hugging, kissing, and sharing of sensitive materials among families were to be avoided altogether, and special greetings at religious gatherings, such as embracing and handshaking, were seriously frowned upon during the peak of COVID-19. As a result, the pandemic in Nigeria has caused many behavioral changes at home, school, and at religious gatherings, as well as in families in general (68–72).

This research examines the influence of MR on SB with evidence from major countries across the world that MR correlatively increased unhealthy SBs and intimate partner violence. We cited various studies that IPV was also aggravated by other co-situations, such as loss of employment and increased demands for time to cater for children caused by a total lockdown. The full understanding of how MR has influenced SB is still unknown. Perhaps more comprehensive knowledge about this nexus will continue to unfold after the COVID era. In the meantime, with the emerging variants of COVID-19, governments have brought back MR at different levels and scales. As a result of the findings, it is recommended that future MR focus on preventing unintended public health failures as denoted in this research. Specifically, partners should be given the opportunity to determine whether they would like to stay together during lockdown or otherwise. Such decisions should be monitored, and partners should be allowed to stay with whoever they choose to stay with and choose how their children will be attended to at such times. This is essential for relationships with a history of violence (73–75). Additionally, government, across different levels, should pay attention to displaced person camps, which apart from their peculiar social and public health challenges (76), may be places where sexual violence is not uncommon. Moreover, people who have been found guilty of sexual violence and related offenses in the past should not be left to cater for young people during lockdown. As a result, stakeholders in sexual and reproductive health, SB, sexually transmitted infections, and intimate partner violence should also concentrate or devote similar attention and resources to every community, notwithstanding the global rating of the level of SB in that country—especially where free movement is being hampered. Finally, this study utilized a relatively small data sample from Owerri Municipal and showed that unhealthy SB exists as a result of MR. It is recommended that a nationwide study of this nature be carried out to see whether similar results would be found. However, this may be a challenge for the government and donor agencies interested in public health and well-being. As the process of MR may have unrooted a prevalent SB problem, it should, nonetheless, be prioritized to create long-lasting health goals, peace, and development for the betterment of society as a whole.

## 5. Conclusion and recommendations

This research examined the influence of MR on SB in a Nigerian context. Evidence has shown across major countries of the world that MR is a correlate of increased unhealthy SBs and intimate partner violence (32, 34, 51, 54, 57, 77). The results of exploring the relationships between certain background characteristics and SB using a chi-squared analysis indicate that age and religious affiliation are the key background variables that have a positive relationship with SB. The regression analysis also showed that MR predisposed young people to certain SBs, such as increased sex chats among themselves, watching erotic movies, pornography, rape, masturbation, incest, and increased distribution of nude pictures. Some of these SBs are considered unhealthy in Africa. Cited from various literature sources, IPV is also aggravated by other co-circumstances, such as loss of work and increased demand for time to cater for children as a direct cause of total lockdown. Nonetheless, the complete picture of how MR has influenced SB is still unknown. More comprehensive knowledge about this nexus will continue to unfold shortly after variants of COVID-19, which are bringing about MRs at different levels and scales, have stopped emerging. As a result of the findings from this study, we recommend the following in case of future MRs so as to prevent unintended public health behavior as described in this research. First, partners should be given the opportunity to determine whether they would like to stay together during a lockdown or otherwise. Such decisions should be monitored, and partners should be allowed to stay with whoever they choose and decide how children will be attended to. This is essential for relationships with a history of violence. Second, people who have been found guilty of sexual violence and related offenses should not be left to cater for young people during a lockdown. Third, governments all over the world should be very careful about how they restrict the movement of young people and design alternatives to cater for their sexual needs in case of future lockdowns to forestall similar unhealthy behavior in the future. Fourth, this study is based on a relatively small sample size in Nigeria. It is recommended that a national study be carried out to better understand whether similar results can be found. Even though this might be a challenge, a nationwide examination of MR on SB will provide valuable information, i.e., cultural and socially, and interested government and donor agencies will obtain a better understanding of how to build a cohesive society in the interest of public health and peace.

## Data availability statement

The original contributions presented in the study are included in the article/Supplementary material, further inquiries can be directed to the corresponding author.

## Author contributions

Conceptualization and writing—original draft preparation: PA. Methodology, software, formal analysis, validation, investigation, resources, and data curation: PA, FU, EA, AO-I, and GC. Writing—review and editing, visualization, supervision, and project administration: GC. All authors have read and agreed to the published version of the manuscript.
