# Supplementary material for: COVID-19, mobility restriction, and sexual behavior among a cohort of people of reproductive age: Nigeria
Source: Front Public Health. 2023 Jan 9;10:935625. doi: 10.3389/fpubh.2022.935625 (PMC9868670; doi:10.3389/fpubh.2022.935625)
Supplement: Supplementary file 1 [file Data_Sheet_1.pdf]

Supplementary Material

COVID-19, mobility restriction, and sexual behavior among a cohort of people of reproductive age: Nigeria

Supplementary Note 1.

➢ ***Questionnaire used in the study***

Questionnaire Number and Coding ___________
Date of Interview ___________

Demography and Social Statistics Program
Department of Economics and Development Studies

QUESTIONNAIRE ON SEXUAL BEHAVIOR DURING THE COVID-19 LOCKDOWN

Dear Respondent,

This is research from the abovenamed Institution and Department. This research will look at the effects of mobility restriction (i.e., lockdown) on sexual behavior among the youth in Owerri, Imo State. The study will enable us to design ways to help youth with healthy sexual behavior in any future lockdowns and related situations. This questionnaire is made up of 40 questions designed to elicit information purely for academic purposes. All information will be treated with utmost confidentiality. Thank you for your anticipated support.

SECTION A: RESPONDENT SOCIO-DEMOGRAPHIC PROFILE: *[Please tick* [✓] *where applicable]*

1. Were you in a relationship when the COVID-19 lockdown began in this town? (i.e., married or dating)?
   Yes [ ], No [ ]
2. State of origin?
   Imo [ ], Enugu [ ], Anambra [ ], Ebonyi [ ], Abia [ ], other [ ]—please specify _______________
3. Indicate which ethnic group you belong?
   Igbo [ ], Hausa [ ], Yoruba [ ], other [ ]—please specify _______________
4. What is your level of formal education?
   No formal education [ ], Primary education [ ], Secondary education [ ], Tertiary education [ ]
5. Please indicate your age group from these categories?
   15-24 [ ], 25-34 [ ], 35-44 [ ], 45+ [ ]
6. Which religion do you belong?
   Christianity [ ], Islam [ ], Traditional religion [ ], other [ ]—please specify _______________
7. What is your sex?
   Female [ ], Male [ ]

8. What is your marital status?
   Single [ ], Married [ ], Divorced [ ], Widowed [ ], Never married [ ], other [ ]—please specify ______________
9. What was your occupation before the lockdown?
   Student [ ], Civil servant [ ], Trading and business [ ], Artisan [ ], Self-employed [ ], Farming [ ], other [ ]—please specify ______________
10. Indicate your monthly income level before the lockdown?
    < ₦20,000 [ ], ₦20,000- ₦39,999 [ ], ₦40,000-₦59,999 [ ], ₦60,000-₦79,999 [ ], ₦80,000+ [ ], None [ ]
11. Are you currently working?
    Yes [ ], No [ ]
12. For how long (i.e., number of months) did lockdown last in this town?
    1-3 months [ ], 4-6 months [ ], 7-9 months [ ], 10+ months [ ], No lockdown [ ]

SECTION B: COMMUNICATION AMONG THE YOUTH DURING COVID-19 LOCKDOWN AND SEXUAL BEHAVIORS

13. How often do you communicate with your partner during the COVID-19 lockdown?
    Everyday [ ], Once in a week [ ], Not frequent [ ]
14. Was there communication breakdown with your partner during the COVID-19 lockdown?
    Yes [ ], No [ ]
15. Common medium of communication with your partner during the lockdown:

		Please tick [✓] all that applies to you or your partner
i.	WhatsApp	
ii.	Telephone and mobile phone	
iii.	Zoom	
iv.	Telegram	
v.	Snapchat	
vi.	Video call	
vii.	Facebook	
viii.	Instagram	
ix.	LinkedIn	
x.	Twitter	
xi.	Deskgram	
xii.	Email	
xiii.	other—please specify:	

16. How long do you spend while communicating during the COVID-19 lockdown?
    Less than 5 minutes [ ], 6-10 minutes [ ], 11-15 minutes [ ], 16-20 minutes [ ], 21+ minutes [ ]
17. What activities did you engage in that was of help to your relationship during the COVID-19 lockdown?
    No activity [ ], Praying for my partner [ ], Watching movies [ ], Regular contact through social media [ ], other [ ]—please specify ______________
18. Did you resort to watching romantic movies or watching sexual contents during lockdown when you are not able to see your lover even after communicating with him/her?
    Yes [ ], No [ ]

19. Did you find a means of satisfying yourself sexually during this mobility restriction period?
    Yes [ ], No [ ]

SECTION C: INFLUENCE OF MOBILITY RESTRICTION ON SEXUAL BEHAVIOUR AMONG THE YOUTH

20. Were you able to go to farm, school, or office during the COVID-19 lockdown?
    Yes [ ], No [ ]
21. Were you able to move about during the COVID-19 lockdown?
    Yes [ ], No [ ]
22. Were you able to hold up sexually all-through the COVID-19 lockdown?
    Yes [ ], No [ ]
23. Do you think you would have been able to hold up sexually if the lockdown was to be extended?
    Yes [ ], No [ ], I doubt it [ ], Maybe [ ]
24. Do you think watching of pornography increased during the lockdown?
    Yes [ ], No [ ]
25. Do you know whether lovers sent nude pictures to one another so as to hold up sexually during the lockdown?
    Yes [ ], No [ ]
26. Did you and you partner or other partners you know practice sex chats during the lockdown?
    Yes [ ], No [ ], I do not know [ ]
27. Did you and your partner try to watch romantic movies during lockdown?
    Yes [ ], No [ ], I do not know [ ]
28. Did you or your partner or anyone you know practice masturbation to hold up sexually during the lockdown?
    Yes [ ], No [ ], I do not know [ ]
29. Did you hear of rape cases during the COVID-19 lockdown?
    Yes [ ], No [ ]
30. If "Yes", do you think this was caused by the lockdown? If "No", go to Question 31.
    Yes [ ], No [ ], I do not know [ ]
31. Did you hear of incest cases during the COVID-19 lockdown?
    Yes [ ], No [ ]
32. If "Yes", do you think this was caused by the lockdown? If "No", go to Question 33.
    Yes [ ], No [ ], I do not know [ ]
33. Do you think people practiced self sex during the COVID-19 lockdown?
    Yes [ ], No [ ]
34. Please tick those sexual behaviors that people of reproductive age practiced during the lockdown:

		Please tick [✓] as appropriate
i.	Sending of nude pictures to each other or watching nude pictures online	
ii.	Watching pornography	
iii.	Masturbating	
iv.	Watching romantic movies	
v.	Sex chats	
vi.	Rape	
vii.	Discussing sex throughout the night	
viii.	Practicing incest (i.e., sex with a brother, sister, cousin, niece, or parents)	

ix.	Practicing self sex	
x.	other—please specify:	

SECTION D: INFLUENCE OF BACKGROUND CHARACTERISTICS ON SEXUAL BEHAVIOUR IN OWERRI

35. Which of the following age categories do you think their sexual behavior will be more affected as a result the COVID-19 lockdown?
    12-19 [ ], 20-27 [ ], 27-35 [ ], 36-40 [ ], 45+ [ ]
36. Do you think the sexual behavior of male youth are more affected than those of female youth as a result of the mobility restriction?
    Yes [ ], No [ ]
37. Which the following educational level do you think is more susceptible to some of the sexual behaviors listed in Question 34 through mobility restriction?
    No formal education [ ], Primary education [ ], Secondary education [ ], Tertiary education [ ]
38. Which of the following marital status is more exposed to adverse sexual behavior during the COVID-19 lockdown?
    Single [ ], Married [ ], Divorced [ ], Widowed [ ], Never married [ ]
39. The sexual behavior of which of the following occupational category is more affected during the mobility restriction?
    Student [ ], Civil servant [ ], Trading and business [ ], Artisan [ ], Self-employed [ ], Farming [ ]
40. Which of the following religious belief do you think will be more affected by practicing any of the listed sexual behaviors in number…above?
    Christianity [ ], Islam [ ], Traditional religion [ ]

END OF QUESTIONNAIRE
THANK YOU FOR YOUR PARTICIPATION

Supplementary Note 2.

- ***Detailed breakdown of the data analysis***

The study utilized three levels of data analysis: univariate, bivariate, and multivariate statistical techniques. Univariate statistical tools were used to present the respondents' background characteristics using percentage distribution and tables. The bivariate analysis examined the relationship between selected socio-demographic characteristics and sexual abstinence during the COVID-19 lockdown in the absence of a spouse. For this part of the study, the basic statistical tool used is the Chi-squared test. The multivariate analysis examined the influence of mobility restriction (MR) on sexual behavior (SB). The dependent variable is measured as a binary code (i.e., No = 0 and Yes = 1), making the response variable amendable to binary logistic regression. Binary logistic regression was used to test the odds of unhealthy SBs influenced by MR. The ability to move about equated to either No or Yes. Binary logistic regression is used to help predict the likelihood of a person engaging in unhealthy sexual gratification as a result of inability to move about (probably) to places where these can be granted. A response of Yes was set as the reference category (i.e., RC) from where other response were referenced. Conversely, the independent variables are SBs which were defined as behavior and activity which are intended to arouse the sexual interest of oneself or another. Different dimensions of SBs have been reported throughout the COVID era in many countries throughout the world. However, eight prominent SBs from the literature formulated the variables of the study and stood out as especially concerning, including: pornography, sending nude pictures, rape, masturbation, watching romantic movies, sex chats, and incest. The analysis adopted 0.05 level of significance which gave a 95% confident interval on the assurance of accepting or rejecting the null hypothesis.

All recovered data from the field were coded and analyzed using the software package SPSS Version 23. The general model for the binary logistic regression utilized Equation 1.

$$Log\left(\frac{p}{(1-p)}\right) = \alpha + \beta_1 X_1 + \beta_2 X_2 + \dots + \beta_n X_n + \dots + e_0 \quad (1)$$

Where: p is the likelihood of being able to move about during lockdown, $1 - p$ is the likelihood of that not to happen, $X_1, \dots X_n$ are a set of independent variables, α is a constant, and β_n are the regression coefficients.

The implicit function of the hypothesis used Equation 2.

$$\delta^{MR} = f(\delta^{SB}) \quad (2)$$

Where: δ^{MR} is the dependent variable measured by being able to move about, and δ^{SB} is the vector which considers the place of the influence MR on different SBs.

The specific variables are presented in Equation 3.

$$\delta^{MR} = f(\delta^{SNP}, \delta^{WP}, \delta^{M}, \delta^{WRM}, \delta^{SC}, \delta^{R}, \delta^{I}, \delta^{SS}) \quad (3)$$

Where: δ^{MR} is the dependent variable measured by being able to move about, δ^{SNP} is sending nude pictures, δ^{WP} is watching pornography, δ^{M} is masturbation, δ^{WRM} is watching romantic movies, δ^{SC} is sex chats, δ^{R} is rape, δ^{I} is incest, δ^{SS} is self sex, and *e* is the error term.

The explicit function of the binary logistic regression formulation is given as Equation 4.

$$\delta^{MR} = \alpha + \beta_1\delta^{SNP} + \beta_2\delta^{WP} + \beta_3\delta^{M} + \beta_4\delta^{WRM} + \beta_5\delta^{SC} + \beta_6\delta^{R} + \beta_7\delta^{I} + \beta_8\delta^{SS} + \dots + e \quad (4)$$

Where: δ^{MR} is the dependent variable measured by being able to move about, δ^{SNP} is sending nude pictures, δ^{WP} is watching pornography, δ^{M} is masturbation, δ^{WRM} is watching romantic movies, δ^{SC} is sex chats, δ^{R} is rape, δ^{I} is incest, δ^{SS} is self sex, β_1 to β_8 are the co-efficient parameters of the independent variables, and *e* is the error term.

Supplementary Note 3.

- ***Additional data on SB***

The deepest cravings in the heart of a human being is the desire to be loved. It is also important to note that love is fueled by communication. One of the things broken by MR, especially if couples were not in the same area when the lockdown was announced, is family communication. For example, there are children who have never lived apart from their parents, i.e., under the same roof, and their communication patterns have developed along this father-mother-child(ren) together pattern. Structurally, Szkody and McKinney (1) point out that family's derive joy and healthy relationships within a household and among friends. The process of the lockdown can psychologically harm this social fabric and increase levels of stress (2–6). What if such children and parents were not locked down in the same place during the COVID-19 restriction? This may not be unconnected with why there were a series of rape cases among adolescents in Nigeria during the lockdown as found in this study. If one is used to receiving affection from parents and loved ones and it suddenly disappears, it may cause abnormal SBs which may make a young lady vulnerable to sexual molestation or exploitation. Research in the United States also found that young adults in romantic relationships enjoy increased daily satisfaction in joint exercise (7), especially early morning jogging or evening workouts. The continuity of some of these physical activities were distorted during the COVID-19 physical distancing rules. People of reproductive age in romantic relationships who are used to regimented exercise may find themselves engaging in unpleasant relationships outside of their partners if they do not enjoy mobility—especially if they are in a different town or region. Women, in particular, may also be lured

or hooked into some unpleasant relationship if they find another person engaging in a similar activity since they are not at home (8–10). This may partly explain the reason why this research finds more women than men, and more specifically separated women, were not able to practice sexual abstinence during MR. A separate study in the United States finds that partners who derive maximum satisfaction through affectionate touch from their partners, report mood swings and stress orchestrated by physical distancing during the COVID-19 MR (11). Imagine what could have happened in the camps of displaced persons in Nigeria during lockdown which are known for sexual harassment and unhygienic conditions even before MR were set in place (12,13). In all these situations, sexual exploitation has gone mostly unreported. This may be partly why Kaukinen (14) and Barbara et al. (15) attributed low reporting of sexual violence to the appropriate authorities in the United States and Italy, respectively.

References

1. Szkody E, McKinney C. Family communication patterns and relationship quality between emerging adults and their parents: *J Soc Pers Relat* (2021) **38**:3177–3197. doi:10.1177/02654075211027217

2. Helland MS, Lyngstad TH, Holt T, Larsen L, Røysamb E. Effects of Covid-19 lockdown on parental functioning in vulnerable families. *J Marriage Fam* (2021) **83**:1515–1526. doi:10.1111/JOMF.12789

3. Johnson MS, Skjerdingstad N, Ebrahimi O V., Hoffart A, Johnson SU. Mechanisms of parental distress during and after the first COVID-19 lockdown phase: A two-wave longitudinal study. *PLoS One* (2021) **16**:e0253087. doi:10.1371/JOURNAL.PONE.0253087

4. Gadermann AC, Thomson KC, Richardson CG, Gagné M, Mcauliffe C, Hirani S, Jenkins E. Examining the impacts of the COVID-19 pandemic on family mental health in Canada: findings from a national cross-sectional study. *BMJ Open* (2021) **11**:e042871. doi:10.1136/BMJOPEN-2020-042871

5. Spinelli M, Lionetti F, Pastore M, Fasolo M. Parents' Stress and Children's Psychological Problems in Families Facing the COVID-19 Outbreak in Italy. *Front Psychol* (2020) **11**:1713. doi:10.3389/FPSYG.2020.01713/BIBTEX

6. Szkody E, Stearns M, Stanhope L, McKinney C. Stress-Buffering Role of Social Support during COVID-19. *Fam Process* (2021) **60**:1002–1015. doi:10.1111/FAMP.12618

7. Sackett-Fox K, Gere J, Updegraff JA. Better together: The impact of exercising with a romantic partner: *J Soc Pers Relat* (2021) **38**:3078–3096. doi:10.1177/02654075211012086

8. Lu JG, Quoidbach J, Gino F, Chakroff A, Maddux WW, Galinsky AD. The dark side of going abroad: How broad foreign experiences increase immoral behavior. *J Pers Soc Psychol* (2017) **112**:1–16. doi:10.1037/PSPA0000068

9. Kincaid C, Zemke DM V. Perceptions of Cheating: An Exploratory Study. *J Hosp Tour Educ* (2013) **18**:47–55. doi:10.1080/10963758.2006.10696849

10. Gilbert D, Abdullah J. A study of the impact of the expectation of a holiday on an individual's sense of well-being: *J Vacat Mark* (2016) **8**:352–361. doi:10.1177/135676670200800406

11. Burleson MH, Roberts NA, Munson AA, Duncan CJ, Randall AK, Ha T, Sioni S, Mickelson KD. Feeling the Absence of Touch: Distancing, Distress, Regulation, and Relationships in the Context of COVID-19: *J Soc Pers Relat* (2021) **39**:56–79. doi:10.1177/02654075211052696

12. Adekola PO, Azuh DE, Amoo EO, Brownell G, Cirella GT. Economic Drivers of Voluntary Return among Conflict-Induced Internally Displaced Persons in Nigeria. *Sustainability* (2022) **14**:2060. doi:10.3390/SU14042060

13. Olanrewaju FO, Olanrewaju A, Omotoso F, Alabi JO, Amoo E, Loromeke E, Ajayi LA. Insurgency and the Invisible Displaced Population in Nigeria: A Situational Analysis. *SAGE Open* (2019) **9**:215824401984620. doi:10.1177/2158244019846207

14. Kaukinen C. When Stay-at-Home Orders Leave Victims Unsafe at Home: Exploring the Risk and Consequences of Intimate Partner Violence during the COVID-19 Pandemic. *Am J Crim Justice* (2020) **45**:668–679. doi:10.1007/S12103-020-09533-5

15. Barbara G, Facchin F, Micci L, Rendiniello M, Giulini P, Cattaneo C, Vercellini P, Kustermann A. COVID-19, Lockdown, and Intimate Partner Violence: Some Data from an Italian Service and Suggestions for Future Approaches. *J Women's Heal* (2020) **29**:1239–1242. doi:10.1089/JWH.2020.8590
